# Diagnosis and Management of Ovarian Tumor in Mayer-Rokitansky-Küster-Hauser (MRKH) Syndrome

**DOI:** 10.1155/2018/2369430

**Published:** 2018-03-12

**Authors:** Yali Miao, Jirui Wen, Liwei Huang, Jiang Wu, Zhiwei Zhao

**Affiliations:** ^1^Department of Obstetrics and Gynecology, Key Laboratory of Birth Defects and Related Diseases of Women and Children of MOE, West China Second University Hospital, Sichuan University, Chengdu, China; ^2^West China School of Preclinical and Forensic Medicine, Sichuan University, Chengdu, China; ^3^West China School of Stomatology Medicine, Sichuan University, Chengdu, China

## Abstract

In the most recent publications on Mayer-Rokitansky-Küster-Hauser (MRKH) syndrome, the uterine remnants and ovaries in patients may develop uterine remnant leiomyoma, adenomyosis, or ovarian tumor, and this can lead to problems in differential diagnosis. Here we summarize the diagnosis methods and available interventions for ovarian tumor in MRKH syndrome, with emphasis on the relevant clinical findings and illustrative relevant case. According to the clinical findings and illustrative relevant case, with the help of imaging techniques, ovarian tumors can be detected in the pelvis in patients with MRKH syndrome and evaluated in terms of size. Laparoscopy could further differentiate ovarian tumors into different pathological types. In addition, laparoscopic surgery not only is helpful for the diagnosis of MRKH combined ovarian tumor, but also has a good treatment role for excising ovarian tumor at the same time. Moreover, laparoscopic removals of ovarian tumor can be considered as a safe and reliable treatment for conservative management.

## 1. Introduction

The Mayer-Rokitansky-Küster-Hauser (MRKH) syndrome is characterized by congenital hypoplasia of the uterus and the upper part of the vagina. The incidence of MRKH syndrome has been estimated as 1 in 4500 women [1]. The principal character is a primary amenorrhea in women presenting with normal development of secondary sexual characteristics and normal external genitalia, but congenital vaginal or a shallow concave nest in the vaginal mouth, congenital uterine, or uterus aplasia. The ovaries are normal and functional as well as the endocrine status. Karyotype is 46, XX, with no visible chromosome modification. At present, most of the studies suggest that MRKH syndrome has been considered as a genetic disease, and genes such as the HOXA7, HOXA9–13, HOXD9–13, and WNT4 have been considered as possible offenders [2].

In the most recent publications on MRKH syndrome, we could found some cases report about uterine remnant leiomyoma, or adenomyosis, but the ovarian tumor is rare in MRKH syndrome and is difficult to be diagnosed [3–8]. Although most publications about pelvic masses in MRKH are about uterine remnants, adenomyosis, or fibroids, the occurrence of ovarian tumors in MRKH could not be ignored as these patients do have ovaries.

Ovarian tumors in patients with MRKH are difficult to examine, especially if no vaginal reconstruction has been performed. The aim of this review is to describe the diagnosis methods and available interventions for ovarian tumor in Mayer-Rokitansky-Küster-Hauser (MRKH) Syndrome, with emphasis on the relevant clinical findings and illustrative relevant case.

## 2. Definition and Prevalence

Mayer-Rokitansky-Küster-Hauser (MRKH) syndrome was first characterized by Mayer, Rokitansky, Küster, Hauser, and Schreiner, which was estimated to complicate 0.2% of births annually [9, 10]. MRKH syndrome is usually present in the form of primary amenorrhea and abnormalities of internal genitalia. But the etiology or pathophysiology of MRKH syndrome is still not well understood. MRKH syndrome has been subdivided into 2 types: type A has isolated Müllerian duct malformations that present as a shallow vaginal dimple with absent cervix, uterus, and upper vagina and is not associated with other anomalies; type B has a similar Müllerian agenesis as type A and also has varying degrees of associated congenital renal malformations (renal agenesis and horseshoe kidney), skeletal abnormalities (scoliosis, spina bifida, and sacral lumenization), and unilateral auditory defects [11].

Ovarian tumors are classified as serous, endometrioid, mucinous, clear cell, and mixed categories, which presents as cystic (single or multicystic), solid cystic, or solid due to diverse structures proportion of cystic and solid fibrotic tissue [12–16]. The presence of ovarian tumors in MRKH patients makes the whole diagnosis and treatment process even more complicated. Although we know more and more about MRKH, the incidence of MRKH with ovarian tumors has not been reported. A review of literature has demonstrated 6 case reports of MRKH syndrome with ovarian tumors (Table 1). After a review of the literature, we find that benign tumors have a majority in the 6 case reports and most studies use laparoscopy to remove the tumors.

## 3. Differential Diagnosis

The differential diagnosis of MRKH syndrome combined pelvic mass mainly includes pelvic mass originated from female genital tract (MRKH syndrome combined uterine fibroids, MRKH syndrome combined uterine adenomyosis, MRKH syndrome combined ovarian tumor, etc.) and derived from other pelvic organ (intestinal tract, mesentery, and retroperitoneal tumor). We need to choose the diagnostic method carefully to differentially diagnose the MRKH syndrome combined pelvic mass. The medical diagnosis of MRKH syndrome is based on the history of primary amenorrhea and the gynecological examination where patients have no vagina and no palpable uterus. The three most common methods of diagnosing MRKH syndrome are by magnetic resonance imaging (MRI), ultrasound, or by laparoscopy.

### 3.1. Illustrative Relevant Case

A 29-year-old young woman, who complained of primary amenorrhea and pelvic mass over 1 year, was used to as the illustrative relevant case. She presented with a cystic pelvic mass 10 cm in diameter on ultrasound and magnetic resonance imaging that could not be differentiated between polycystic ovary and ovarian cystadenoma. The patient was laparoscopically operated on, and the left ovarian tumor was detected and removed. Histology confirmed a benign ovarian serous cystadenofibroma.

### 3.2. Imaging Features

**Figure 1:**
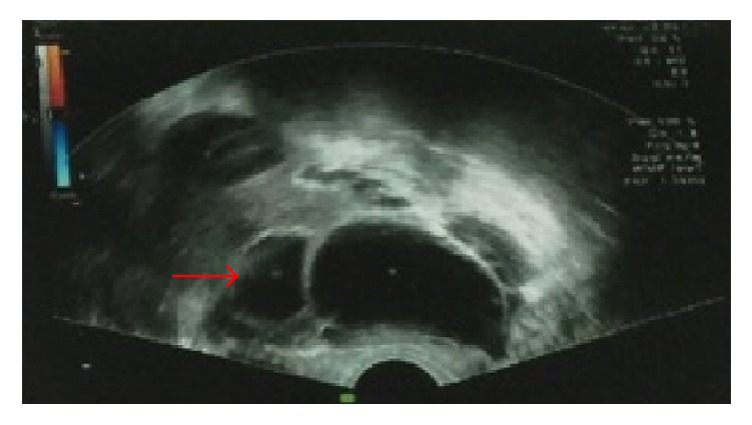
A 29-year-old female patient with primary amenorrhea. Transvaginal ultrasound shows no uterine; red arrow: polycystic ovary tumor with clear boundary.

According to the literature review, ultrasound, MRI, and CT are the major imaging tools to diagnose the MRKH syndrome with ovarian tumors. The ultrasound image of MRKH syndrome is characterized by no normal uterus to be found in either longitudinal or cross-cutting image in the back of the filling bladder, but normal volume ovary on both sides [17]. Moreover, it is essential to check the abdominal cavity and the groin area to find the heterotopic uterus, ovary, and urinary system malformation by transabdominal ultrasound. Therefore, Ultrasonography is the most basic test for patients with MRKH syndrome and is helpful in finding the ovarian lesions. The method of ultrasonography plays an important role in the preliminary diagnosis [18]. But for surgical interventions, ultrasound may not always be effective in finding Müllerian buds and ovaries, which is an important factor when deciding on which method of surgery is best for an MRKH patient [11]. From our illustrative relevant case, with transvaginal ultrasound (Figure 1), 10.9 cm × 9.2 cm× 7.4 cm heterogeneous mass below the bladder was noticed, but no uterus was found. There were many small anechoic areas in the large mass. The mass boundary was clear and cyst wall was smooth; the ovarian tissue surrounded the cyst, in which there were no blood flow and no pelvic effusion. Liver, spleen, bile cyst, pancreas, and kidney were normal.

**Figure 2:**
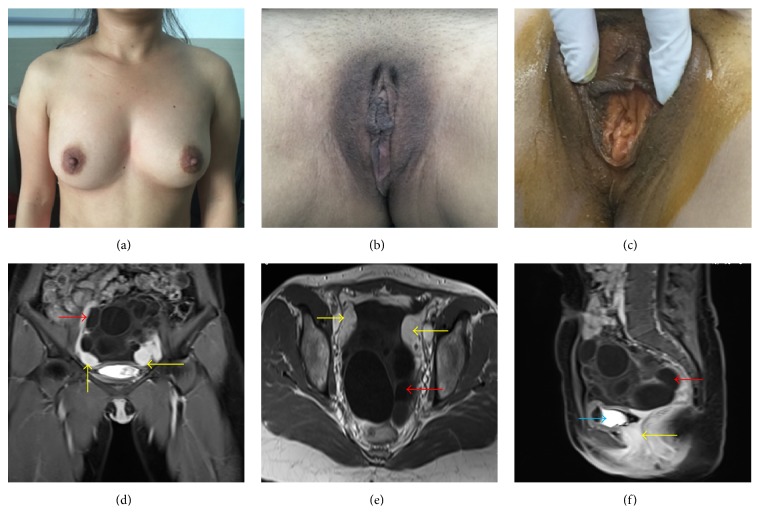
A 29-year-old female patient with primary amenorrhea. (a) Well developed breasts. (b) Vulva. (c) Vaginal vestibule. (d) T1W1 coronary view: yellow arrow: bilateral primordial uterus and red arrow: ovary tumor. (e) T1W1 axial view: yellow arrow: bilateral primordial uterus and red arrow: ovary tumor. (f) T1W1 sagittal view: yellow arrow: vagina, blue arrow: bladder, and red arrow: ovary tumor.

MRI is helpful in identifying pelvic mass derived from ovaries or from intestinal tract, mesentery, and retroperitoneal tumor. American College of Obstetricians and Gynecologists (ACOG) suggested that the initial diagnosis of MRKH syndrome should be combined MRI scans to identify possible abnormalities in the patient [19]. MRI could check the malformation of reproductive system, urinary system, and skeletal system sensitively. MRI diagnosis of MRKH syndrome was 100% sensitive and specific as confirmed by laparoscopy [20]. MRI can depict the distance of an obstructed vagina from the perineum and the thickness of a vaginal septum or atretic segment. MRI examination can accurately measure the gap between the neck and rectum of the urethra and provide references for the selection of surgical methods [21]. However, MRI could confirm the pelvic mass derived from ovaries but could not distinguish between polycystic ovary and multilocular ovarian serous cystadenoma. From our illustrative relevant case, with MRI (Figure 2), it showed two solid nodules at bilateral accessory area but showed no normal uterine morphology; the diameter of solid nodules was less than 3 cm, which showed slightly long* T*1 and* T*2 signal, equivalent DWI signal, and obvious homogeneous enhancement after enhancement scanning. In two solid nodules there was no clear endometrial morphology signal. Between two masses there was strengthening cord above the vagina top. In the pelvic cavity there were multiple size differential oval cysts with long* T*2,* T*1 signal, and clear boundary; the largest cyst was about 4.5 cm × 5.6 cm × 4.5 cm. The multiple oval cysts showed DWI strong signal, and no low signal of ADC or enhanced edge was found after enhancement scanning. There were no enlarging pelvic lymph nodes.

MRI and ultrasound imaging are valuable tools to diagnose the MRKH syndrome, as well as evaluate patients for concurrent renal anomalies, endometrioma, and tubal disease. Although CT is not a common method of diagnosing MRKH syndrome, it should be kept in mind that CT is useful in finding the ovarian tumors in MRKH syndrome.

From the above, imaging tools as ultrasound, MRI, and CT are valuable to find the ovarian tumors in MRKH syndrome and distinguish it from intestinal tract, mesentery, and retroperitoneal tumor. However, it is hard for imaging tools to diagnose the pathological type of ovarian tumors before operation.

### 3.3. Laparoscopy Features

**Figure 3:**
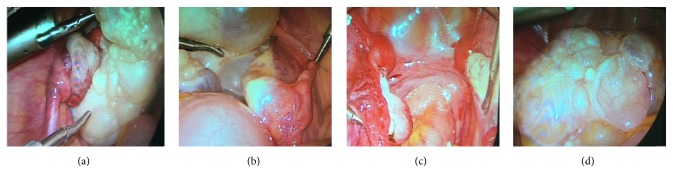
(a) The right attachment was 180 degrees of torsion. (b) The right attachment. (c) After left ovarian neoplasm resection. (d) Left ovary tumor.

**Figure 4:**
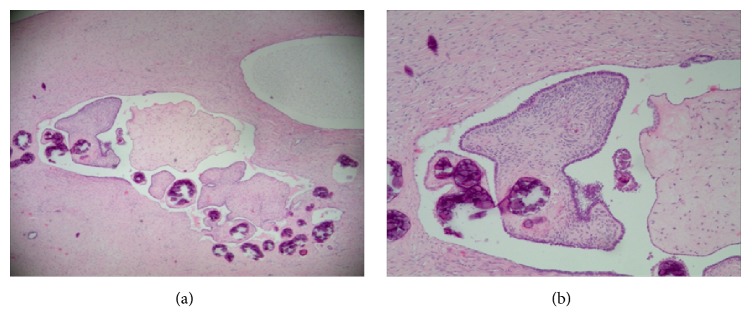
A 29-year-old female patient with primary amenorrhea. (a) Microscopic findings of H&E staining (×10). (b) Microscopic findings of H&E staining (×40). Morphology of calcification in ovarian cancer.

Laparoscopy is the gold standard for evaluation of MRKH syndrome. But laparoscopy used in MRKH diagnosis is not an attractive method due to its invasive nature. Laparoscopy is more expensive than MRI and should be reserved for patients undergoing surgical intervention or guiding the process [11]. For MRKH patients with ovarian tumors, laparoscopy offers the possibility of diagnosing and treating at the same time. From our illustrative relevant case, laparoscopy was performed and revealed a large pelvic mass about 10 cm in diameter, which was located in left ovary lateral margin and was a multiple cystic clear boundary ovarian tumor with complete capsule. Suspensory ligament of left ovary, left proper ligament of ovary, and fallopian tube were 180 degrees of torsion. We thought the reason of the tumor torsion is associated with the heterogeneity of tumor. Left fallopian tube was normal, connected with left uterine nodules. The right attachment was normal, connected with right solid nodules (Figure 3). The complete resection of the left ovary tumor was taken along the left ovary pole, resetting the left ovary and the fallopian tube. The pathological examination showed ovarian serous papillary cystadenofibroma (CAF) (Figure 4).

## 4. Treatment

Laparoscopy is the ideal technique to identify and treat ovarian benign tumor, so it may also be able to treat the ovarian benign tumor in MRKH syndrome. From our illustrative relevant case, we treated a rare case of large ovarian serous papillary cystadenofibroma in a young woman with the MRKH syndrome with laparoscopic surgery. After 6 months of postoperative follow-up, the patients recovered well. To the best of our knowledge, our illustrative relevant case describes the fifth case in which ovarian tumor in MRKH syndrome was removed under laparoscopy confirming that laparoscopy is a powerful tool for treatment as well as diagnosis of these tumor. In addition, cytoreductive surgery and oophorectomy are further needed to treat the ovarian malignant tumor in MRKH syndrome.

## 5. Conclusion

With the help of imaging techniques, ovarian tumors can be detected in the pelvis in patients with MRKH syndrome and evaluated in terms of size. Laparoscopy could further differentiate ovarian tumors into different pathological types. In addition, laparoscopic surgery not only is helpful for the diagnosis of MRKH combined ovarian tumor, but also has a good treatment role for excising ovarian tumor at the same time. Moreover, laparoscopic removals of ovarian tumor can be considered as a safe and reliable treatment for conservative management. From the above, we think women should be inspected regularly, especially adolescents without menstruation, to check genital tract malformation and discover the pelvic diseases, such as ovarian tumors, leiomyoma, and attachment mass.

## Figures and Tables

**Table 1:** Case reports of MRKH syndrome with ovarian tumors.

Study	Published year	Study location	Age	Diagnosis methods	Pathological type	Treatment
Fukuda et al. [22]	2010	Japan	50	MRI, laparotomy and histological analysis	Ovarian mucinous cystadenoma	Laparoscopic resection
Huepenbecker et al. [23]	2017	United States	64	CT, laparotomy and histological analysis	Serous ovarian adenocarcinoma	Laparoscopic resection
Juusela et al. [24]	2017	United States	72	Laparotomy and histological analysis	Bilateral ovarian Sertoli cell tumors	Laparoscopic resection
Mishina et al. [25]	2007	Moldova	35	Ultrasound and histological analysis	Ovarian dysgerminoma	Oophorectomy
Nusrath et al. [26]	2016	India	65	CT, laparotomy and histological analysis	Ovarian endometrioid carcinoma	Laparoscopic resection and cytoreductive surgery
Tsaur et al. [27]	1995	China	4	Ultrasound, CT and histological analysis	Ovarian teratoma	Oophorectomy

MRI: magnetic resonance imaging; CT: computed tomography.
